# Successful surgical in situ treatment of prosthetic graft infection by staged procedure after Bentall operation and total aortic arch replacement

**DOI:** 10.1186/2193-1801-3-172

**Published:** 2014-04-02

**Authors:** Paschalis Tossios, Avgerinos Karatzopoulos, Konstantinos Tsagakis, Konstantinos Sapalidis, Konstantina Triantafillopoulou, Anna Kalogera, Georgios T Karapanagiotidis, Vasilios Grosomanidis

**Affiliations:** Department of Cardiothoracic Surgery, Aristotle University Medical School, AHEPA University Hospital, Thessaloniki, Greece; Department of Thoracic and Cardiovascular Surgery, West German Heart Center, University Hospital Essen, Essen, Germany; 3rd Surgical Department, Aristotle University Medical School, AHEPA University Hospital, Thessaloniki, Greece; Department of Radiology, Aristotle University Medical School, AHEPA University Hospital, Thessaloniki, Greece; Department of Anesthesia and Intensive Care, Aristotle University Medical School, AHEPA University Hospital, Thessaloniki, Greece

**Keywords:** Prosthetic graft infection, Ascending aorta, Aortic arch, Omentum transposition, In situ preservation

## Abstract

We report a case of a 29-year-old Marfan patient who developed prosthetic graft infection 10 months after Bentall operation and successive replacement of the remaining ascending aorta and the entire aortic arch for acute aortic dissection. Instead of an aggressive high-risk aortic redo procedure with removal and replacement of the infected prosthetic graft we elected a staged graft-sparing surgical approach. After 18 months of close follow-up the patient is in good condition and free from infectious sequela. This case and our review of the literature suggest that open extensive disinfection followed by tissue flap coverage is highly effective in controlling thoracic aortic prosthetic graft infection and may be considered as first-line treatment in such high-risk aortic arch redo patients.

## Background

There is no consensus on proper surgical management for prosthetic graft infection in patients who have had an operation for reconstruction of ascending aorta and aortic arch. Historically, a more aggressive but high-risk aortic redo procedure with removal and replacement of the infected prosthetic graft has been proposed by individual surgeons in order to control this condition, with unsatisfactory results (Coselli et al. 1990; Coselli et al. 1999). However, this type of surgery cannot be performed in all patients. In recent years, an increasing body of published evidence suggests that a more conservative, nonresectional, graft-sparing surgical strategy with open surgical disinfection followed by omentum flap coverage of the infected ascending and arch prosthetic graft is gaining wider acceptance as a more feasible and more effective treatment therapy with excellent immediate and midterm outcomes (Coselli et al. 1990; Coselli et al. 1999; Hargrove and Edmunds 1984; Nakajima et al. 1999; LeMaire and Coselli 2007). This case report contributes the ongoing discussion on this important topic and may help to pay cardiac surgeons’ attention on modern knowledge-based treatment strategies, and also thus avoid in selected patients unnecessary high-risk aortic redo procedures for infected ascending and arch prosthetic grafts.

## Case description

**Figure 1:**
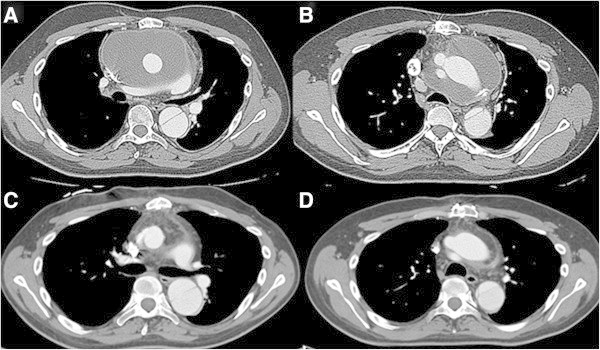
**Multi-slice computed tomography (CT) of the chest.** Preoperative CT shows a huge mediastinal fluid collection surrounding both **A)** the ascending, and **B)** aortic arch prosthetic grafts having a diameter of 15 cm with a low density (12-18 Hounsfield Units). Postoperative CT shows omental wrapping with normal density (65-80 Hounsfield Units) around the **C)** the ascending, and **D)** aortic arch prosthetic grafts without evidence of fluid collection or infectious sequela 18 months after the operation.

**Figure 2:**
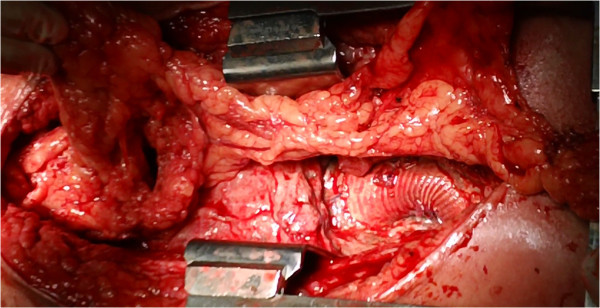
**Intraoperative photograph.** A pedicle of omentum is brought up into chest to fill the mediastinal cavity and to surround the exposed prosthetic graft.

A 29-year-old man with Marfan syndrome was referred for assessment of a huge mediastinal fluid collection associated with an aortic prosthetic graft. At age 20 years, he had undergone Bentall procedure with a short-cut composite graft (Carbomedics Inc., Austin, TX) for acute aortic dissection type A. Eight years later, he had a successive replacement of ascending aorta and the entire aortic arch with a 24-mm Dacron graft (Gelweave, Vascutek Ltd, Renfrewshire, Scotland), again for acute aortic dissection involving the remaining aorta Surgery was performed under moderate hypothermic circulatory arrest with selective antegrade cerebral perfusion. The repair was accomplished by ligation of the left subclavian artery and extranatomic reconstruction with an aorto-subclavian bypass by placing an 8-mm vascular Dacron graft. The patient was extubated at the first postoperative day (POD), and subsequently was transferred to the ward. Intra- and peri-operative antibiotic prophylaxis consisted by ciprofloxacin and daptomycin as the patient had known drug allergies to penicillin and cephalosporin antibiotics. The patient was discharged home on POD 12. He was well and fit until ten months later. At that moment he presented to his local hospital with hyperexia (39.5°C), sweats, and chills. Routine laboratory investigations revealed a leukocytosis of 19 × 10^9^ cells/L. The patient was started on intravenous rifampin and daptomycin. Multi-slice computed tomographic (CT) aortography of his chest demonstrated evidence of a huge fluid collection surrounding both the ascending and aortic arch prosthetic grafts. Transthoracic echocardiography and subsequent transesophageal echocardiography confirmed the above findings. No prosthetic valve malfunction or valvular vegetation was found and the blood cultures were negative. Five days after his initial presentation, the patient was transferred to our institution for further care. The patient was afebrile and hemodynamically stable. Upon patient’s physical examination no surgical site infection was present. Chest roentgenogram revealed a soft infiltration of the left lower lobe. Repeat CT angiography was performed the next day to definitively evaluate the integrity of anastomotic sites. There was no evidence of anastomotic leak or false aneurysm formation. The collection had a diameter of 15 cm with a density not exceeding 20 Hounsfield Units strongly suggestive of prosthetic graft infection (Figure 1 A-B). The patient underwent subsequently fine-needle aspiration of purulent fluid under CT guidance which confirmed the clinical diagnosis of prosthetic graft infection that needed to be treated. Two hours after the intervention, the patient was brought to the operating room and the median sternotomy incision was reopened. Surgical exploration of the mediastinum demonstrated a considerable amount of purulent fluid with which the ascending/arch prosthetic graft was flooded. Suction of turbid effusion was followed by moderate debridement of necrotic, infected tissues around the prosthetic graft. Suture lines were intact but exposed within the contaminated field. The aortic root, however, was entirely covered with healthy autogenous tissue and thus not exposed to pus. In the initial step, the mediastinal cavity, especially around the graft, was thoroughly irrigated and washed initially with 3 liters of diluted, 1% iodine solution, and then with 1 liter of saline solution containing 1 g vancomycin. Thereafter, sponges soaked with undiluted, 10% iodine solution were packed around the contaminated graft as well as in the surrounding operative field. The patient was returned to intensive care unit (ICU) with the chest left open but covered by aseptic drape. The same procedure of irrigation and packing of the mediastinum was repeated every 8 hours for a 48 hour period in the ICU, while the patient was maintained intubated and sedated. The patient was brought back to the operating room for the second operative step 48 hours later. The omentum was harvested via a short upper abdominal midline incision, separately from the previous sternotomy wound, and transferred as a vascularized pedicle into the mediastinal cavity in order to wrap around the ascending and arch prosthetic graft and anastomotic sites with omentum completely (Figure 2). The chest was closed in layers without placement of chest tubes or irrigation catheters. The patient had no operative complications, completed four weeks of intravenous colistin, teicoplanin, and metronidazole therapy, and was thereafter discharged home in good condition on a regimen of orally administered moxifloxacin. Cultures of serial blood samples as well as specimens obtained both directly from the infection site under CT guidance and intraoperatively from the perigraft pus collection were negative throughout the course of treatment. The patient remains well without evidence of infectious sequela 18 months after the operation (Figure 1 C-D).

## Discussion

Although prosthetic graft infections after ascending and/or arch surgery are very rare (<3%), surgeons should remain cognizant of them because of the potential morbidity and mortality complication (Coselli et al. 1990). There are no surgical guidelines, and treatment of ascending and arch prosthetic graft infection is still challenging (Teebken et al. 2012). Traditionally, many authors believe that removal and in situ replacement of all the prosthetic material with a new one is mandatory. However, surgical mortality and morbidity are still a major concern with traditional replacement due to the invasiveness and complexity of the procedure, emergency conditions, contaminated field, and the difficulties of exposure in the redo aortic setting (Czerny et al. 2011). Two recent explants studies from dedicated aortic centers, to our knowledge the largest published series, have reported a hospital mortality which ranges from 24% to 27% (Coselli et al. 1999; Khaladj et al. 2013). These have been reports of small series, including 11 patients from Texas and 17 patients from Hanover in which redo ascending aortic replacement and/or aortic arch replacement were performed by using Dacron grafts or homografts without recurrences. The leading cause of mortality was multiple organ failure following sepsis or profound bleeding (Coselli et al. 1999; Khaladj et al. 2013). Successful redo in-situ replacement surgery in this series was even among early survivors associated with severe postoperative complications such as wound infection, respiratory failure, stroke, and multiple organ failure (Coselli et al. 1999). However, other authors including our group with solid practice abroad experienced exceptionally disappointing results following the principle of aggressive redo ascending/arch prosthetic graft replacement, due to uncontrollable infective process (Hargrove and Edmunds 1984; Nakajima et al. 1999). As a result, many institutions switched during the last decades to a more conservative, nonresectional, graft-sparing surgical approach which appears to have become a method of choice under certain circumstances (Coselli et al. 1990; Coselli et al. 1999; Hargrove and Edmunds 1984; Nakajima et al. 1999; LeMaire and Coselli 2007; Akowuah et al. 2007). We have reviewed 77 cases of any type of in situ preservation for the treatment of infected ascending aortic and/or aortic arch prosthetic grafts reported in the literature to date and found a collective early survival rate of 95% and a 100% success rate in terms of nonreccurrence of graft infection (Coselli et al. 1990; Coselli et al. 1999; Hargrove and Edmunds 1984; Nakajima et al. 1999; LeMaire and Coselli 2007; Akowuah et al. 2007). These excellent results are based on retrospective analyses of surgical cohorts and case reports including patients with ascending aortic grafts, many of whom also received an arch graft, composite valve graft, or separate aortic valve prosthesis (Coselli et al. 1990; Coselli et al. 1999; Hargrove and Edmunds 1984; Nakajima et al. 1999; LeMaire and Coselli 2007; Akowuah et al. 2007). In our case, graft-sparing ascending aortic and aortic arch redo surgery in the presence of an additional extranatomic aorto-subclavian Dacron graft was performed in a patient without prosthetic aortic valve endocarditis or prosthetic valve dysfunction. The staged approach was a safe and effective treatment strategy and preferable to conventional replacement surgery as it is less invasive due to the avoidance of the stress of an open replacement with anticoagulation for cardiopulmonary bypass, thus lessening the risk for fatal bleeding or uncontrollable sepsis. Furthermore, short-term disinfection period with open irrigation and packing of the entire infected spaces helps eradicate and sterilize the complete operating field prior to omentum flap coverage of the infected prosthetic graft (Nakajima et al. 1999). Major advantages of omentum flap include the rich of blood supply contributing to control the infection, sufficient volume to fill the mediastinal cavity, aortic coverage, and ease to perform (Coselli et al. 1990; Coselli et al. 1999; Hargrove and Edmunds 1984; Nakajima et al. 1999; LeMaire and Coselli 2007). Particularly with regard to durability all the published cases have specifically documented the absence of recurrent graft infection after the definitive in situ preservation procedure. Nearly all of the reports included follow-up of at least two years. In some cases nonreccurrence of graft infection has been reported up to 10 years after such aortic redo procedures (Nakajima et al. 1999).

## Conclusions

Nonresectional, in situ graft-sparing surgical therapy is safe and effective in patients with ascending and arch prosthetic graft infection, as long as the infection is not associated with native or prosthetic aortic valve endocarditis or valve dysfunction, demonstrating superior outcomes in comparison with traditional redo aortic replacement. The recognition of this clinical issue has the potential to spare patients unnecessary high-risk aortic redo operations. However, there are no definitive treatment recommendations for ascending and arch prosthetic graft infection. Clearly, more cases like this need to be reported and monitored over time to support this conclusion.

## Consent

Written informed consent was obtained from the patient for the publication of this report and any accompanying images.

## Abbreviations

CT: Computed tomography
ICU: Intensive care unit
POD: Postoperative day.
